# Haematophagous ectoparasites lower survival of and have detrimental physiological effects on golden eagle nestlings

**DOI:** 10.1093/conphys/coab060

**Published:** 2021-08-09

**Authors:** Benjamin M Dudek, Michael T Henderson, Stephanie F Hudon, Eric J Hayden, Julie A Heath

**Affiliations:** 1 Department of Biological Sciences and Raptor Research Center, Boise State University, Boise, ID 83725, USA; 2 The Peregrine Fund, Boise, ID 83709, USA; 3 Department of Biological Sciences, Biomolecular Sciences Graduate Program, Boise State University, Boise, ID 83725, USA

**Keywords:** telomere, parasitism, Mexican chicken bug, Haematosiphon inodorus, corticosterone, Aquila chrysaetos

## Abstract

Haematophagous ectoparasites can directly affect the health of young animals by depleting blood volume and reducing energetic resources available for growth and development. Less is known about the effects of ectoparasitism on stress physiology (i.e. glucocorticoid hormones) or animal behaviour. Mexican chicken bugs (*Haematosiphon inodorus*; Hemiptera: Cimicidae) are blood-sucking ectoparasites that live in nesting material or nest substrate and feed on nestling birds. Over the past 50 years, the range of *H. inodorus* has expanded, suggesting that new hosts or populations may be vulnerable. We studied the physiological and behavioural effects of *H. inodorus* on golden eagle (*Aquila chrysaetos*) nestlings in southwestern Idaho. We estimated the level of *H. inodorus* infestation at each nest and measured nestling mass, haematocrit, corticosterone concentrations, telomere lengths and recorded early fledging and mortality events. At nests with the highest levels of infestation, nestlings had significantly lower mass and haematocrit. In addition, highly parasitized nestlings had corticosterone concentrations twice as high on average (42.9 ng/ml) than non-parasitized nestlings (20.2 ng/ml). Telomeres of highly parasitized female nestlings significantly shortened as eagles aged, but we found no effect of parasitism on the telomeres of male nestlings. Finally, in nests with higher infestation levels, eagle nestlings were 20 times more likely to die, often because they left the nest before they could fly. These results suggest that *H. inodorus* may limit local golden eagle populations by decreasing productivity. For eagles that survived infestation, chronically elevated glucocorticoids and shortened telomeres may adversely affect cognitive function or survival in this otherwise long-lived species. Emerging threats from ectoparasites should be an important management consideration for protected species, like golden eagles.

## Teaser

The Mexican chicken bug (*Haematosiphon inodorus*)*,* a blood-sucking ectoparasite, may present a new threat to some birds. Golden eagle nestlings parasitized by *H. inodorus* are in poorer physiological condition and grow more slowly than non-parasitized nestlings. Parasitism increases the probability that nestlings will fledge early, often leading to death.

## Introduction

Parasites can have deleterious effects on animals from diverse life histories and geographic locations (Møller *et al.*, 2009). Parasite virulence varies widely among systems, but as the ranges of parasites shift in response to changes in landscape and climate, naive hosts that lack physiological and behavioural defence mechanisms will be exposed to novel challenges (Cumming and Van Vuuren, 2006; Møller *et al.*, 2013; Cable *et al.*, 2017). Haematophagous ectoparasites survive on blood meals from their hosts and can negatively affect individual survival, particularly of young animals (Merino and Potti, 1995). For altricial bird species, nestlings are particularly vulnerable to the negative effects of haematophagous ectoparasites because they are unable to escape infested nests during a time of high energetic demands, rapid structural growth and maturation of physiological systems (Dube *et al.*, 2018). Understanding how ectoparasites, particularly novel species, influence nestling condition, development and physiology is important for the conservation and management of avian species because costs incurred during growth and development may have long-term negative effects on host survival and population abundance (Møller, 1993; Brown *et al.*, 1995).

The costs of parasitism are important to hosts when they lower reproductive success or survival (Newton, 1998). Haematophagous ectoparasites siphon nutritional resources from hosts by consuming blood meals (Lehmann, 1993) and severe infestations can adversely affect nestling growth, induce anaemia and lead to haemorrhages and muscle weakness (Brown and Brown, 1986; Chapman and George, 1991; Justice-Allen *et al.*, 2016). Further, exposure to ectoparasitism may elicit a physiological stress response leading to elevated corticosterone (Quillfeldt *et al.*, 2004; Eggert *et al.*, 2010). Chronically elevated corticosterone can have significant costs for developing birds, such as reduced growth efficiency (Müller *et al.*, 2009), compromised immune defence (Stier *et al.*, 2009) and decreased cognitive ability (Kitaysky *et al.*, 2003). Finally, the effects of ectoparasitism on nestlings may be reflected in telomeres, the repetitive DNA sequences on the ends of chromosomes. Telomeres can be prematurely shortened by parasitic infections, which could lead to cellular senescence, altered tissue function and adverse effects on organismal health (Asghar *et al.*, 2016). Further, relationships between parasitism and telomere length may be sex specific because of endocrine–immune interactions (Sudyka *et al.*, 2019). Shorter telomeres in early life, as well as faster rates of telomere shortening over time, have been shown to predict longevity (Bize *et al.*, 2009; Heidinger *et al.*, 2012; Tricola *et al.*, 2018) and reproductive success (Pauliny *et al.*, 2006; Eastwood *et al.*, 2019) in different avian species. Therefore, telomere lengths may provide additional information on the effects of ectoparasitism on nestling fitness and survival.

The Mexican chicken bug (*Haematosiphon inodorus*; Hemiptera: Cimicidae) is an avian haematophagous ectoparasite that affects North American raptor species (Lee, 1955; Platt, 1975; Grubb *et al.*, 1986). Mexican chicken bugs live in nest material and surrounding substrate and require blood meals during every stage of life (Usinger, 1966). McFadzen *et al.* (1996) reported on the northern range expansion of *H. inodorus* into southwestern Idaho, but little is known about the distribution and abundance of *H. inodorus* in relation to nesting raptors in North America. Although few studies have considered the ecological impact of *H. inodorus* on raptors, McFadzen and Marzluff (1996) found that prairie falcon (*Falco mexicanus*) nestlings infested by *H. inodorus* in southwestern Idaho had lower mass and haematocrit levels and experienced mortality as a result of high infestation levels. This finding is similar to what has been reported in other avian species parasitized by cimicids (Brown and Brown, 1986).

Golden eagles (*Aquila chrysaetos*) are a widespread, but uncommon, large-bodied raptor currently facing threats across their North American range (Kochert and Steenhof, 2002). High densities of golden eagles nest along the cliffs and rocky outcrops near the Snake River in southwestern Idaho (Steenhof *et al.*, 1997). Golden eagle nests often contain a diverse assemblage of ectoparasites (Katzner *et al.*, 2020), and the presence of *H. inodorus* has been documented in eagle nests in southwestern Idaho (McFadzen *et al.*, 1996), but the physiological consequences of *H. inodorus* infestation on eagle nestlings have not been studied. Understanding how *H. inodorus* affect golden eagle nestlings is important for the conservation and management of the species, which faces threats across its range from urbanization and human population growth (Kochert and Steenhof, 2002). Our objective was to assess the effect of *H. inodorus* infestation on nestling eagle health. Specifically, we examined whether the level of *H. inodorus* infestation affected nestling mass, haematocrit levels, corticosterone concentrations and telomere lengths. In addition, we assessed the relationship between *H. inodorus* infestation and the probability of nestlings departing nests before they could fly, often resulting in mortality, or dying in the nest.

## Materials and methods

### Study area

**Figure 1:**
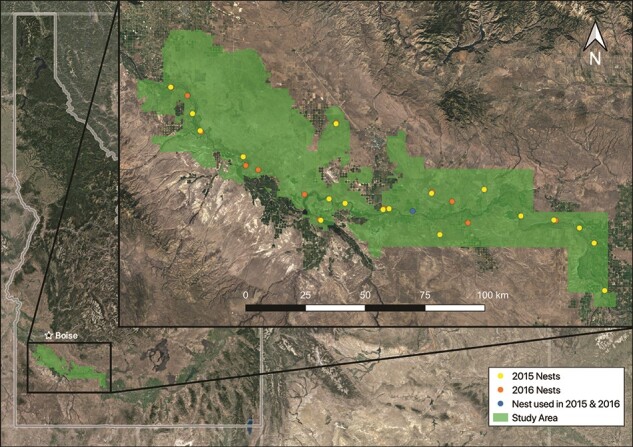
Location of study area (represented by green polygon) in southwestern Idaho, USA. Yellow circles represent golden eagle nests sampled in 2015; orange circles, golden eagle nests sampled in 2016; and the single blue circle, a nest used in both 2015 and 2016.

Our study area was located in southwestern Idaho along the Snake River Canyon in, and adjacent to, the Morley Nelson Snake River Birds of Prey National Conservation Area (NCA; Fig. 1). Steep basalt cliffs in the Snake River Canyon provide ample ledges for golden eagles to construct large stick nests. Golden eagle nesting territories in southwestern Idaho often contain multiple alternative nests (Millsap *et al.*, 2015. The number of nests per territory can range from 1 to 18 with some pairs using the same nest every year, whereas other pairs switch nests within their territory from year to year (Kochert and Steenhof, 2012). The surrounding uplands in the NCA consist of native shrub-steppe and salt-desert communities characterized by sagebrush-dominated shrublands, native perennial grasses, disturbed grasslands and rangeland dominated by exotic annual grasses, irrigated agricultural land and rural and suburban development (U.S. Department of the Interior, 1996).

### Field techniques

In 2015 and 2016, we monitored 35 historical golden eagle nesting territories to determine occupancy and breeding attempts following the methods described in Steenhof *et al.* (1997). We made regular nest observations from >400 metres and visually assessed nestling age based on feather development (Driscoll, 2010). We began visiting nests every 8–10 days when nestlings were able to thermoregulate (ca. 21 days old; Katzner *et al.*, 2020) until nestlings were ~51 days old (typically 3 or 4 visits). After nestlings reached 51 days, we monitored nestlings from remote observation points every 3–5 days to determine survival and age at fledging. If fledging was not directly observed, fledging age was estimated as the average between the last date the nestling was observed in the nest and the first date the nestling was not observed in the nest. If a nestling left the nest before they were 51 days old (80% of average fledging age; Steenhof *et al.*, 2017), we categorized that nestling as an ‘early’ fledgling because typically these birds were not able to fly, and we searched the area underneath the nest for recently fledged young or carcasses. We continued to monitor recent fledglings until they moved away from the immediate nest area (~400 metres), at which point determining their location and fate became increasingly difficult.

During each nest visit, we visually assessed the abundance of adult and nymph *H. inodorus* present on the nest surface and on nestlings for 4–8 minutes and grouped infestation levels into three categorical ranks: (1) no infestation, no bugs observed in the nest material or on nestlings; (2) low infestation. 1–100 bugs observed in total; or (3) high infestation, >100 bugs observed. In a related study, these infestation categories correlated with quantitative estimates of *H. inodorus* density made from pit fall traps placed in the nest (Dudek, 2017). We recorded nestling mass using a Pesola spring scale during each visit and categorized the fullness of nestlings’ crops into quartiles. We collected blood samples from the brachial vein when nestlings were ~28 and 49 days old with a 25-gauge needle and syringe. We collected blood for haematocrit and genetic sexing in both years and collected blood for corticosterone in 2015 and telomeres in 2016. During blood-collection visits, we used a cloth sack to transport nestlings from the nest to the top of the cliff where we worked in teams to collect blood. In 2015, we recorded the duration of time between our first contact with nestlings and the completion of blood sampling to account for the researcher-induced stress response on corticosterone in statistical models (Romero and Reed, 2005) and attempted to collect samples within 20 minutes. At some nests we were unable to collect blood within 20 minutes and we did not measure corticosterone. Blood samples for corticosterone assays were collected into 0.8 ml lithium heparin mini centrifuge tubes. For haematocrit samples, blood was aliquoted into two heparinized 75 mm capillary tubes per nestling. In 2016, we used the packed blood cells from haematocrit samples to measure telomere lengths. All blood samples were kept on ice in the field until processing at Boise State University on the same day of sampling. We centrifuged haematocrit samples for 6 minutes at 11000 × g and measured the proportion of packed blood cells relative to whole blood. Haematocrit levels were represented by the average of the two samples. For the corticosterone samples, we centrifuged blood samples at 6048 × g for 10 minutes to separate plasma from the cellular fraction of whole blood and stored plasma samples and cells separately at −20°C. We stored whole blood cells in lysis buffer for telomere analysis in 2016 and to determine nestling sex with single-nucleotide polymorphism genotyping (Doyle *et al.*, 2016) at Purdue University (West Lafayette, IN, USA; 2015 samples) or by Avian Biotech International (Tallahassee, FL, USA; 2016 samples). All field methods followed protocols approved by the Boise State University Institutional Animal Care and Use Committee (Protocol #006-AC14-007).

### Corticosterone assay

We used enzyme-linked immunosorbent assays (ELISA, Cayman Chemicals) to estimate corticosterone concentrations in nestling plasma. We divided each plasma sample into 30-μl duplicates then twice extracted corticosterone using 5 ml of diethyl ether. We poured off the lipophilic supernatant and evaporated it under a stream of nitrogen gas in a warm water bath. Following kit instructions, we reconstituted extracted samples with 100 μl of buffer, vortexed and divided into 50-μl aliquots that were added to 96-well plates coated with mouse monoclonal antibody. We added corticosterone-specific acetylcholinesterase tracer and rabbit corticosterone antiserum to the 96-well plates and then incubated plates on an orbital shaker for 2 hours. We then rinsed plates to remove any non-bound corticosterone and developed them in a dark chamber with Ellman’s reagent for 1 hour. We read plates at 405 nm with a Biotek EL800 plate reader. We validated the corticosterone assay by comparing the slopes of a plasma dilution curve to the assay standard curve slope. Plasma dilution curves consisted of different ratios of pooled eagle plasma to buffer. There was no significant difference between the slopes of the plasma dilution and assay standard curve. We calculated the concentration of corticosterone in samples by comparing results to a standard curve established with known concentrations. We determined extraction efficiency by analysing a standard corticosterone sample and calculated inter-assay variation from repeated values of a pooled sample. We corrected all values for assay extraction efficiency (mean ± SD) 88.0 ± 9.4%. Inter-assay variation averaged 5.4%, and average intra-assay variation was 2.7%.

### Telomere assay

We extracted DNA using the Zymo Quick-DNA Microprep Plus Kit (#D4074) according to the manufacture’s protocol. We assessed DNA purity by 260/280 nm absorbance ratio (NanoDrop). We quantified DNA by absorbance at 260 nm, and the concentration was normalized to 2 ng/μl in an elution buffer containing 10 mM Tris-HCl pH 8.5 and 0.1 mM EDTA (Zymo). We performed Quantitative PCR (qPCR) using a Roche LC96. We performed PCR reactions in 20-μl volumes containing ~8 ng DNA, 10 μl of 2x Biotium Fast Plus EvaGreen® qPCR Master Mix and 10 pmol each of forward and reverse primers (500 nM final primer concentration). A standard curve for each primer pair was included in triplicate on each plate. We prepared the standard curve by seven serial dilutions (1∶4) of DNA. We used the standard curve to determined DNA concentrations of each sample using both the reference and telomere primers. We quantified telomere lengths as the ratio of the DNA concentration determined with the telomere primers divided by the average of the concentrations determined from the two reference primers (Hudon *et al.*, 2020; Table 1). Telomere primers are previously published (Cawthon, 2002). For all primers, we used a two-step thermal cycling profile of 95°C for 2 minutes, followed by 40 cycles of 95°C for 5 seconds and 55°C for 30 seconds, with signal acquisition at the end of the 55°C step, followed by a melt curve generated by increasing temperatures at the end of the thermal cycling. We ran all samples in triplicate along with three negative controls per primer pair. If the variation between technical replicates (Ct standard deviation) was greater than 0.5, we repeated the assay for that sample.

**Table 1:** Reference gene and telomere primer sequences used to estimate the length of telomere-DNA sequences of golden eagle nestlings in southwestern Idaho, USA, in 2016

Name	Sequence
tel 1b	CGGTTTGTTTGGGTTTGGGTTTGGGTTTGGGTTTGGGTT
tel 2b	CAGCCGAAAGGCCCTTGGCAGGAGGGCTGCTGGTGGTCTACCCTT
UCE.28-F	AAATACCACCCAACAGTTT
UCE.28-R	AAGCCCTATACAGATGGAT
UCE.239-F	TCAGATGTTCAGCCTATT
UCE.239-R	AATACCATGTTAATTATCCTCAA

### Statistical analysis

We used linear mixed models (LMMs) to examine the effects of *H. inodorus* infestation on nestling mass, haematocrit, corticosterone concentrations, telomere length and the probability that nestlings would fledge early or die. We included nest identity as a random variable in all models and nestling identity as a random variable in the nestling mass, haematocrit, corticosterone concentrations and telomere length models. Sampling year was included as a random variable in the nestling mass, haematocrit and the probability that nestlings would fledge early or die models that were sampled in 2015 and 2016, but not for the corticosterone or telomere length models that were only measured in 1 year. We treated *H. inodorus* infestation level as a category to account for possible non-linear effects of infestation of physiology, with the exception of the model to explain whether nestlings fledged early or died where *H. inodorus* infestation level was treated as a numerical rank. To examine effects on nestling mass and haematocrit, we used the following fixed effects: *H. inodorus* infestation level at the time of sampling, age (in days, linear and polynomial), sex, the interaction between infestation level and age and the interaction between infestation level and sex. Before modelling, we adjusted mass for crop contents using a sex- and age-specific crop size equation to estimate the mass of crop contents (Collopy, 1984) and then subtracted crop mass from measured body mass. We used a pairwise comparison to evaluate the mean differences between each infestation level. We used an LMM to predict the effect of age, sex, time of day, handling time (minutes), infestation level at the time of sampling and the interaction between infestation level and handling time on nestling corticosterone concentrations. We used an LMM to examine whether age, sex, infestation level at the time of sampling or interactions among these factors, explained telomere length. Finally, we created a generalized LMM with a binomial distribution to predict the probability that nestlings in highly infested nests fledged early or died. We used the infestation level when nestlings were approximately 28 days old (mean 28.5 ± 3.1 days) for this analysis because we did not have infestation levels at the time nestlings died or fledged early. No nestlings died or left the nest before 28 days, so the infestation level at 28 days could be applied across all birds in our study. Finally, we compared whether there were differences in mass, haematocrit and telomere length for nestlings that fledged early or died and nestlings that fledged successfully at >51 days with an LMM that included nestling identity, nest and year (for mass and haematocrit) as a random effect. We did not make this comparison for CORT because we had a low sample size for the fledged early or died group. All analyses were performed in R (version 4.0.0, R Core Team, 2020) and linear models were created using functions lmer and glmer in the package lme4 (Bates *et al.*, 2015). Interactions were removed if they were not significant. Pairwise comparisons were conducted using the function lsmeans in the package lsmeans (Lenth, 2016). Descriptive statistics are reported as mean ± standard deviation and confidence intervals (CIs) are reported at 95%.

## Results

We measured *H. inodorus* infestation levels at occupied nests in 19 and 16 golden eagle territories in 2015 and 2016, respectively. Ten territories were sampled in both years (different nests in each year) and one nest was used 2 years in a row and thus was sampled in both years. Of the 35 nests sampled, 23% (*n* = 8) experienced no *H. inodorus* infestation, 46% (*n* = 16) experienced low levels of infestation and 31% (*n* = 11) experienced high levels of infestation.

**Figure 2:**
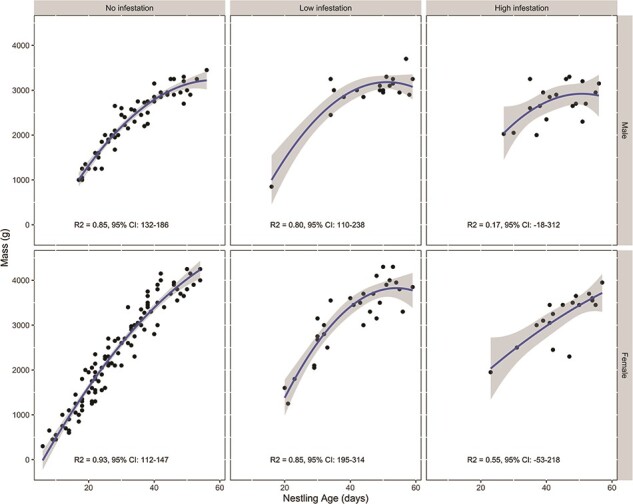
Golden eagle nestling mass (black circles), predicted mass (blue line) and associated 95% CIs (solid grey area) measured from nestlings experiencing different levels of *H. inodorus* infestation in nests in southwestern Idaho, USA, in 2015 and 2016. The R^2^ and 95% CIs for the effect of age on mass is shown at the bottom of each grid cell. Nestlings in the low- and high-infestation group gained mass more slowly as they developed compared to nestlings in the no-infestation group (χ^2^ = 13.5, *P* < 0.01).

We measured nestling mass from 31 eagle nestlings in 2015 and 26 nestlings in 2016. There was a significant interaction between age and *H. inodorus* infestation rank on mass (χ^2^ = 13.50, *P* < 0.01; Fig. 2) and females were significantly heavier than males (χ^2^ = 73.71, *P* < 0.01). Nestlings in the no-infestation and low-infestation groups tended to gain mass as they grew (all 95% CI for effect of age positive) with the rate of mass gain tapering as they reached mature sizes (Fig. 2); however, nestlings in the low-infestation group had significantly lower rate of mass gain compared to the no infestation group (Table A1). Nestlings in the high-infestation group had a lower rate of mass gain compared to the no-infestation group (Table A1), and in females, the 95% CIs for effect of age overlapped zero suggesting that female nestlings may not gain mass during growth in this group (Fig. 2). There was no interaction between age, the level of *H. inodorus* infestation and sex (interaction term χ^2^ = 0.27, *P* = 0.87).

**Figure 3:**
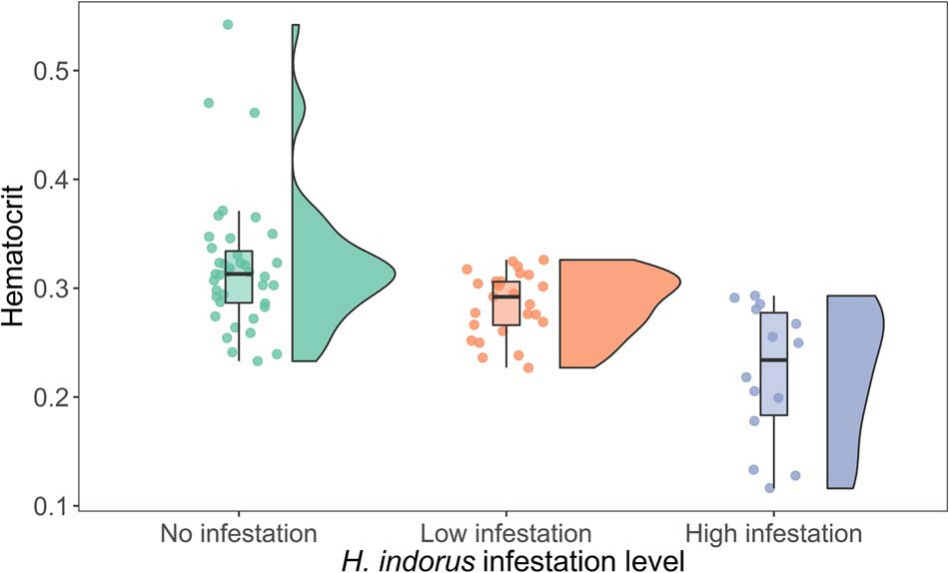
Haematocrit measured from golden eagle nestlings experiencing different levels of *H. inodorus* infestation in nests in southwestern Idaho, USA, in 2015 and 2016. Bold lines within boxes represent the median, upper and lower limits of the box are the first and third quartiles, whiskers contain 1.5 times the interquartile range and the coloured circles represent each measured value. The width of the shaded area in the violin plot represents the distribution of haematocrit values. Nestling haematocrit decreased in highly infested nests (χ^2^ = 25.1, *P* < 0.01). Asterisk indicates a statistically different group.

We measured haematocrit from 24 nestlings in 2015 and 26 nestlings in 2016. Mean nestling haematocrit over both seasons was 0.29 ± 0.06 (range, 0.12–0.54). Nestlings from nests with high levels of *H. inodorus* infestation had significantly lower haematocrit than nestlings from non-infested nests or nests with low levels of infestation (χ^2^ = 19.2, *P* < 0.01; Fig. 3; Table A2). Haematocrit did not vary by nestling age (χ^2^ = 1.05, *P* = 0.30) or sex (χ^2^ < 0.1, *P* = 0.97).

**Figure 4:**
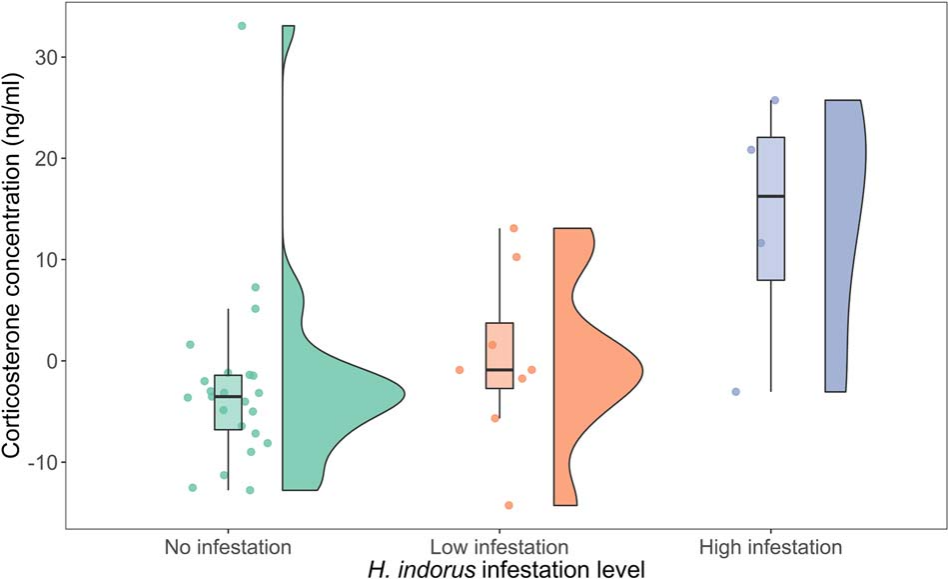
Residual corticosterone concentrations from a model that included handling time (minutes) for golden eagle nestlings experiencing different levels of *H. inodorus* infestation in nests in southwestern Idaho, USA, in 2015. Bold lines within boxes represent the median, upper and lower limits of the box are the first and third quartiles, whiskers contain 1.5 times the interquartile range and the coloured circles represent each measured value. The width of the shaded area in the violin plot represents the distribution of residual corticosterone concentration. Nestling corticosterone concentrations (ng/ml) increased in highly infested nests (χ^2^ = 21.1, *P* < 0.01). Asterisk indicates a statistically different group.

Circulating corticosterone concentrations were measured from 26 nestlings in 2015. Nestling corticosterone concentrations averaged 22.29 ± 15.17 ng/ml (range, 3.70–80.65 ng/ml). Corticosterone concentrations were significantly higher for nestlings that experienced high *H. inodorus* infestation compared to nestlings from nests with either no infestation or low infestation (χ^2^ = 26.7, *P* < 0.01; Fig. 4; Table A3). Nestling handling time was 9.7 ± 3.2 minutes (range, 4.0–19.0). Nestling corticosterone concentrations increased with handling time (β = 2.53, 95% CI = 1.23, 3.78, χ^2^ = 17.5, *P* < 0.01), but there was no significant interaction between handling time and infestation level (χ^2^ = 2.6, *P* = 0.28), suggesting that infestation category did not influence the acute stress response. We found no effect of nestling age (χ^2^ = 0.4, *P* = 0.51), sex (χ^2^ < 0.1, *P* = 0.90) or time of day (χ^2^ = 0.3, *P* = 0.60) on corticosterone concentrations.

**Figure 5:**
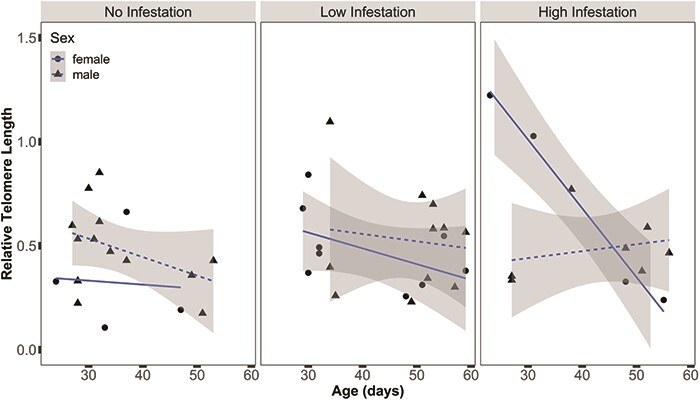
Observed golden eagle nestling telomere lengths (female, black circles; male, black triangles), predicted length (female, solid blue line; male, dotted blue line) and associated 95% CIs (solid grey area) measured from nestlings experiencing different levels of *H. inodorus* infestation in nests in southwestern Idaho, USA, in 2016. Female nestling telomere lengths decreased in highly infested nests (χ^2^ = 8.9, *P* = 0.01). For females in the high-infestation group, R^2^ = 0.98; for all other groups with no relationship over time, R^2^ < 0.1.

We quantified telomere lengths for 26 nestlings in 2016. Telomere lengths ranged from 0.11 to 1.22. We found a significant interaction between sex, age and infestation level on telomere lengths (χ^2^ = 8.9, *P* < 0.01). Telomeres of female nestlings significantly shortened with age in highly infested nests, whereas telomeres of female nestlings did not change in nests with either no infestation or low infestation nests. Male nestling telomere length did not change with age or infestation level (Fig. 5; Table A4).

**Figure 6:**
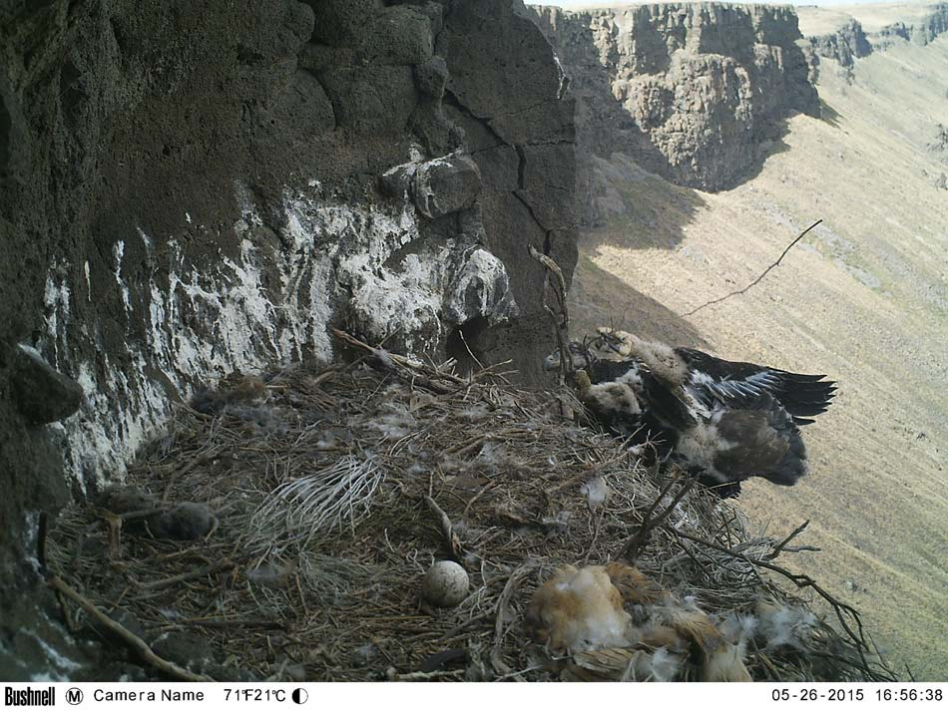
A golden eagle nestling, ~56 days old, falling to its death from a nest highly infested with *H. inodorus* in southwestern Idaho, USA in 2015. This photo was captured by a motion-activated camera installed on the cliff wall adjacent to the nest.

We used data from 29 nestlings in 2015 and 25 nestlings in 2016 to assess effect of *H. inodorus* on the probability of nestlings leaving the nest before 51 days of age or dying in, or below, the nest. Nestlings in our study left the nest at 62 ± 8 days (range, 42–74 days) on average. About a quarter of the nestlings, 22% (12/54), died in the nest prior to fledging, left the nest before 51 days old (45 ± 3 days) or fell out of the nest and died shortly after reaching fledging age. Three nestlings were found dead in the nest prior to fledging, seven nestlings fledged early, five of which were found dead below the nest and two ‘fledge-age’ nestlings (aged 55 days and 56 days) fell out of the nest and were found dead below the nest. We found that *H. inodorus* infestation level at a nest was positively associated with the probability that nestlings left the nest early or died (β = 25.8, 95% CI = 7.6, 44.2; χ^2^ = 7.76, *P* < 0.01; Table 2, Fig. 6). There was no significant difference in mass between nestlings that left the nest early or died (Fig. A1; χ^2^ = 0.7, *P* = 0.41). Nestlings that left the nest early or died had lower haematocrit than nestlings that lived and fledged at a later age (Fig. A2; χ^2^ = 9.9, *P* < 0.01). Corticosterone concentrations of nestlings that left the nest early or died were higher (64.2 ± 11.2 ng/ml, *n* = 2) compared to corticosterone concentrations of nestlings that lived and fledged at a later age (19.7 ± 11.3 ng/ml, *n* = 25). There was no significant difference in telomere lengths between eagles that lived and eagles that died or left the nest <51 days (Fig. A3; χ^2^ = 0.2, *P* = 0.64).

**Table 2:** The count and percentage of golden eagle nestlings that fledged from the nest before 51 days or died for each rank of *H. inodorus* infestation in southwestern Idaho, USA, in 2015 and 2016

		Nestlings that fledged early or died in the nest	
Infestation level	Total number of nestlings	Number	Percentage
No infestation	42	3	7%
Low infestation	7	6	86%
High infestation	5	5	100%

## Discussion

Ectoparasitism by *H. inodorus* had a detrimental effect on golden eagle nestling health and survival, suggesting *H. inodorus* may be an emerging or understudied threat to golden eagles in southwestern Idaho. The effects of higher levels of *H. inodorus* infestation on nestling mass and haematocrit suggest that *H. inodorus* ectoparasitism created an energetically expensive cost to nestling development and physical condition. Additionally, corticosterone concentrations were higher in nestlings exposed to high levels of *H. inodorus* infestation and telomere lengths of female nestlings were significantly shorter in highly infested nests, suggesting that *H. inodorus* ectoparasitism was physiologically stressful. Finally, *H. inodorus* infestations were positively associated with early fledging, which can lead to death for young birds leaving cliff nests before they are capable of flight, and mortality prior to fledging. We found 22% of eagle nestlings in our study fledged early or died prior to fledging in nests with high levels of *H. inodorus* infestation. Taken together, these results indicate that *H. inodorus* infestation contributed to reduced productivity through nestling mortality and may have lasting effects on young eagles that survive the nestling phase.

High ectoparasite infestations can negatively affect fitness-related traits in birds (Møller, 1990; Hurtrez-Boussès *et al.*, 1997; Heeb *et al.*, 2000). There is a positive relationship between nestling mass (body condition) and juvenile survival (Arizaga *et al.*, 2015; Jones *et al.*, 2017), so events that birds experience as nestlings may have long reaching effects. Similar to other studies (e.g. Whitworth and Bennett, 1992; Hurtrez-Boussès *et al.*, 1997), we found that nestling haematocrit decreased in highly infested nests, suggesting a direct effect of ectoparasitism on nestling condition. The reported haematocrit range for healthy raptors is 0.35–0.55 (Monks and Forbes, 2007); however, nestlings typically have lower haematocrit values than adults (reviewed in Fair *et al.*, 2007). In our study, nestling haematocrit ranged from 0.12 to 0.54 and high levels of *H. inodorus* ectoparasitism were associated with the lowest haematocrit values.

Anaemia and metabolic stress can cause immunosuppression and increase the susceptibility of birds to pathogenic agents (Folstad and Karter, 1992). During development, nestling birds make trade-offs between growth, survival and immune function (Brommer, 2004). Although *H. inodorus* is not a known vector of any pathogen, the closely related swallow bug (*Oeciacus vicarius*) is a vector for the Buggy Creek virus (Togaviridae: Alphavirus) and transmits the pathogen to cliff swallows (*Petrochelidon pyrrhonota*; Fassbinder-Orth *et al.*, 2013). Additionally, Justice-Allen *et al.* (2016) reported that parasitism by Argasid ticks caused paresis and ataxia in bald eagle (*Haliaeetus leucocephalus*) nestlings. Avian tick paralysis is caused by neurotoxins associated with many tick species (Luttrell *et al.*, 1996). Although ectoparasitism decreased the physiological condition of nestlings, we noted no other abnormal effects of *H. inodorus* ectoparasitism on eagle nestlings and repeated biting did not appear to cause additional symptoms. Further study is needed to evaluate the potential role of *H. inodorus* as a vector of arboviruses and other diseases.

Our study is the first to report circulating corticosterone concentrations for golden eagles and we validated the use of ELISA assays on eagle plasma. Our results show that the intensity of *H. inodorus* infestation influences corticosterone concentrations in eagle nestlings and suggest that glucocorticoids act as a mechanism to overcome the effects of ectoparasitism. Ectoparasitism has been linked to elevated corticosterone concentrations in some bird species (Quillfeldt *et al.*, 2004; Raouf *et al.*, 2006), but not others (Lobato *et al.*, 2008; Eggert *et al.*, 2010; Pryor and Casto, 2015), suggesting that an adrenal response to ectoparasitism may depend on the ability of the host species to cope with the parasitism event (St. Juliana *et al.*, 2014). Interspecific differences in stress response to ectoparasitism demonstrate the importance of investigating corticosterone response in raptor species. The pattern of exploitation by the ectoparasite and the history of association between the host and ectoparasite may explain differences in host responses. Host species that co-evolve with parasite species may exhibit less of a stress response due to acquired physiological defence mechanisms (Khokhlova *et al.*, 2008). Conversely, relatively new host–parasite relationships may allow parasites to exert a comparatively strong negative effect on their hosts (Koop *et al.*, 2013). Therefore, the stress response elicited by golden eagle nestlings in our study to *H. inodorus* infestation may be related to the relatively recent arrival of *H. inodorus* in southwestern Idaho.

Chronically elevated corticosterone caused by ectoparasitism could be deleterious to the survival of eagle nestlings by reducing immune function (Wingfield *et al.*, 1997) and cognitive capabilities (Kitaysky *et al.*, 2003). Corticosterone has been found to stimulate activity in numerous avian species including dispersal in juvenile western screech-owls (*Megascops kennicottii*; Belthoff and Dufty, 1998), pre-migratory restlessness in red knots (*Calidris canutus*; Piersma *et al.*, 2000) and migratory flights of bar-tailed godwits (*Limosa lapponica*; Landys-Ciannelli *et al.*, 2002), and nestling corticosterone concentrations have been shown to increase prior to fledging (Heath, 1997; Corbel and Groscolas, 2008). Given the positive effect that corticosterone has on movement (Breuner *et al.*, 1998), it is possible that increased corticosterone concentrations, resulting from high *H. inodorus* infestation, may have facilitated fledging attempts before nestlings were able to fly. A controlled study that accounts for confounding effects of reduced provisioning near the time of fledging (Bustamanté and Hiraldo, 1990), energetic deficits caused by parasites and other stressors would be necessary to test this hypothesis. Unfortunately, we did not have these conditions or the sample size to evaluate this relationship.

In our study, we recorded nine instances of early or unsuccessful fledging from highly infested nests, 78% of which resulted in mortality. Early fledging from nests with high *H. inodorus* infestations has been documented in prairie falcons (McFadzen and Marzluff, 1996) and golden eagles (Morales-Yañez and Rodríquez-Estrella, 2019). Increased movements within a cliff nest’s limited area may increase the risk for individuals experiencing deteriorating health conditions. At one nest in 2015, a 56-day-old nestling, previously observed to be in poor physical condition, was documented falling off the edge of a highly infested nest to its death. Furthermore, decreased cognitive ability as a result of high corticosterone concentrations can reduce foraging ability of recently fledged birds, which could increase the likelihood of post-fledging mortality due to starvation (Kitaysky *et al.*, 2003). Although we do not know the long-term fate of all nestlings in our study, we observed that some nestlings from highly infested nests survived to fledge and disperse from their natal territory. The long-term effects of low mass and haematocrit on the survival of fledgling eagles are unknown and warrant further study.

We found that the telomeres of female nestlings, but not males, shortened with age when exposed to high infestations of ectoparasites. Sex-specific effects of parasites on telomere dynamics has also been observed in Eurasian blue tits (*Cyanistes caeruleus*) and may reflect hormonal mechanisms that mediate the response to parasitic infections in male birds (Klein, 2004; Sudyka *et al.*, 2019). Alternatively, eagles exhibit reverse sexual-size dimorphism and the high cost of infestation on growth rates (mass gain) may have been more severe for larger female nestlings.

Given the costs of ectoparasitism to nestling condition, development and survival, it is important to understand the physiological response to ectoparasite infestation. When avian populations are stressed by habitat reduction or alteration, the individual response to haematophagous ectoparasites may compound negative population-level effects (Loye and Carroll, 1998). North American golden eagle populations, like many wildlife populations worldwide, continue to face threats from habitat loss and the reduction of their historical prey populations (Kochert and Steenhof, 2002). Our study was not an experimental study and therefore we cannot rule out that *H. inodorus* infestation levels are correlated with other factors that may explain the physiological effects we observed. Increased monitoring and study of the effects of ectoparasitism on the condition and survival of young animals through experimental studies, especially in systems experiencing shifts in parasite distributions and the introductions of novel parasite species associated with climate change, will be important for future conservation efforts of imperilled species.

## Funding

This work was supported by the U.S. Fish and Wildlife Service Western Golden Eagle Team (Project numbers F15AP00200 and F16AC00242), Bureau of Land Management (Project number L14AC00342), Boise State University Raptor Research Center, a National Science Foundation Research Experiences for Undergraduates award (DBI-1263167 to M.T.H.) and an National Science Foundation Track 2 EPSCoR Program award (OIA-1826801).

## Conflicts of Interest

None declared.

## Supplementary Material

coab060_APPENDIX
