# Supplementary material for: Haematophagous ectoparasites lower survival of and have detrimental physiological effects on golden eagle nestlings
Source: Conserv Physiol. 2021 Aug 9;9(1):coab060. doi: 10.1093/conphys/coab060 (PMC8354271; doi:10.1093/conphys/coab060)
Supplement: coab060_APPENDIX [file coab060_appendix.docx]

**APPENDIX**

Figures showing the relationships between health metrics (nestling mass, hematocrit, and telomere length) and whether or not golden eagle nestlings fledged early or died in the nest.


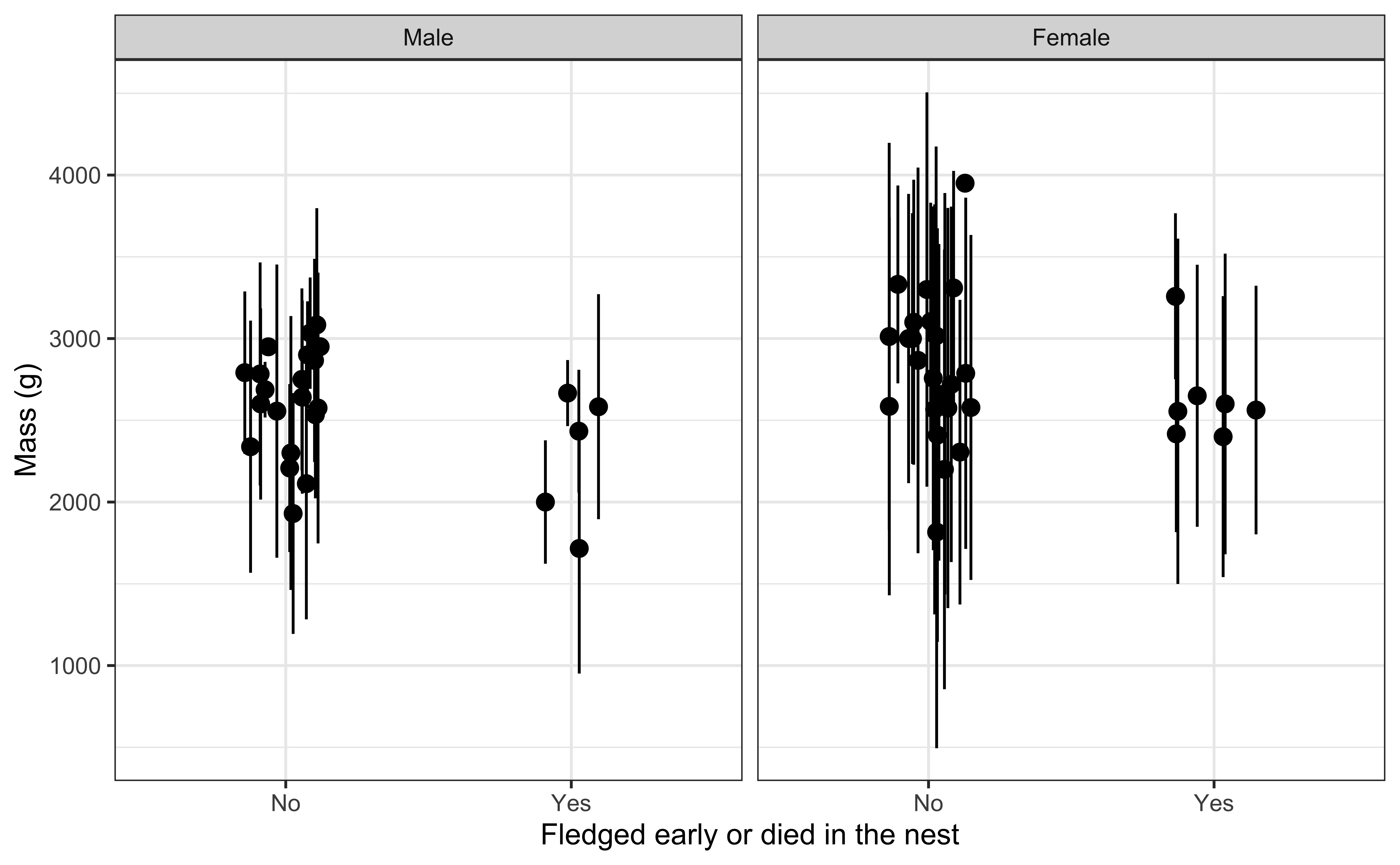


Figure A1. The relationship between mass and whether or not a nestling golden eagle fledged before 51 days or died in the nest for males and females. There was no significant difference in mass between eagles that lived and eagles that died or left the nest < 51 days (χ^2^ = 0.7, *P* = 0.41). Each point represents mean mass for one bird and the error bars represent standard deviation.


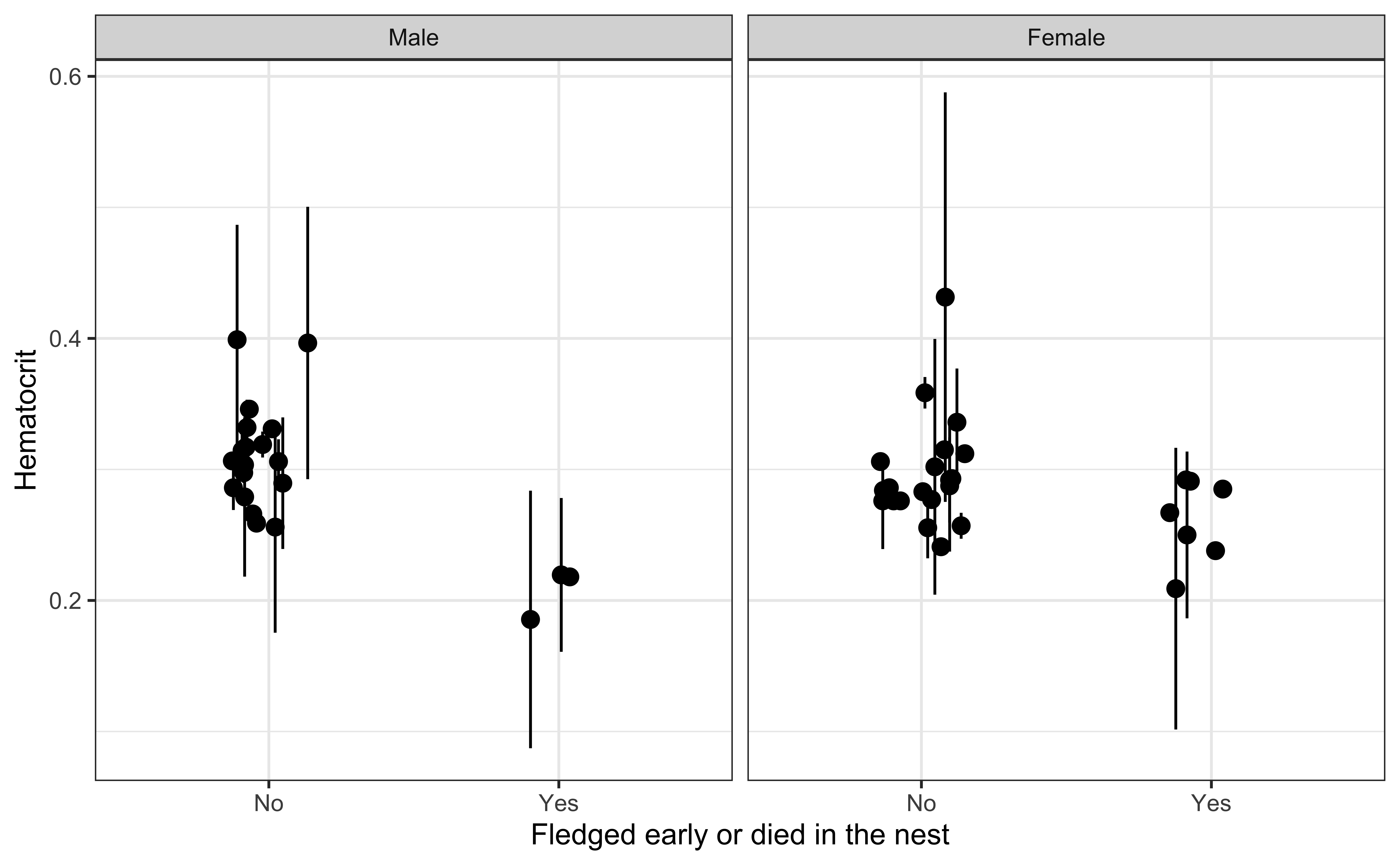


Figure A2. The relationship between hematocrit and whether or not a golden eagle fledged before 51 days or died in the nest for males and females. Nestlings that left the nest early or died had lower hematocrit that nestlings that lived and fledged at a later age (χ^2^ = 9.9, *P* < 0.01). Each point represents mean hematocrit for one bird and the error bars represent standard deviation. Points with no bars show values for birds that were only sampled once.


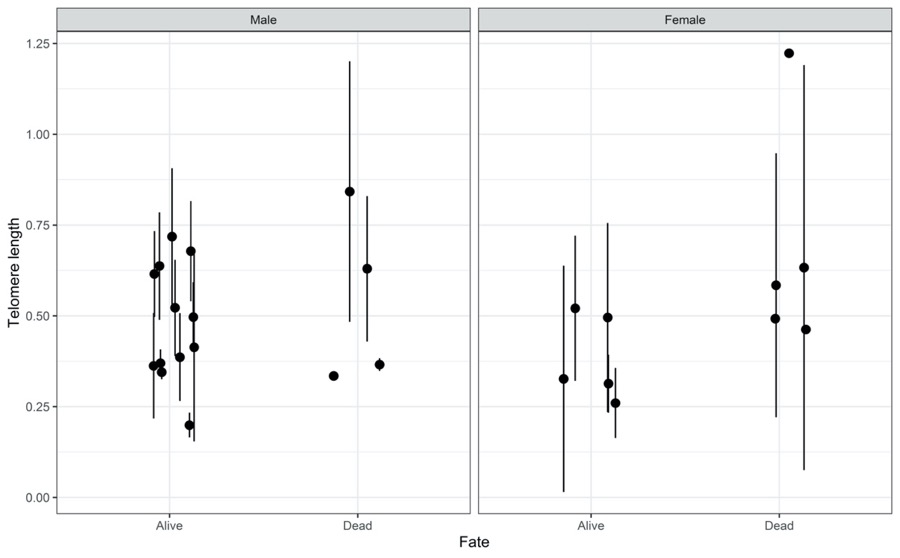


Figure A3. The relationship between telomere length and whether or not a golden eagle survived to 51 days or died for males and females. There was no significant difference in telomere lengths between eagles that lived and eagles that died or left the nest < 51 days (χ^2^ = 0.2, *P* = 0.64). Each point represents mean telomere length for one bird and the error bars represent standard deviation. Points with no bars show values for birds that were only sampled once.

Table A1. Table of golden eagle nestling mass slope comparisons (rate of mass gain as eagles age) at each level of *H. inodorus* infestation in nests in southwestern Idaho, USA in 2015 and 2016. An asterisk (*) indicates a statistically significant difference.

| Comparison | Estimate | Std Error | t-value | P-value |
| --- | --- | --- | --- | --- |
| Low infestation - No infestation == 0 | -12.77 | 4.52 | -2.82 | < 0.01* |
| High infestation - No infestation == 0 | -20.11 | 6.44 | -3.12 | < 0.01* |
| High infestation - Low infestation == 0 | -7.34 | 6.61 | 1.11 | 0.31 |

Table A2. Means comparisons of golden eagle nestling hematocrit at each level of *H. inodorus* infestation in nests in southwestern Idaho, USA in 2015 and 2016. An asterisk (*) indicates a statistically significant difference.

| Comparison | Estimate | Std. Error | z value | Pr(>\|z\|) |
| --- | --- | --- | --- | --- |
| Low infestation - No infestation == 0 | -0.02 | 0.01 | -1.53 | 0.13 |
| High infestation - No infestation == 0 | -0.08 | 0.02 | -4.38 | < 0.01* |
| High infestation - Low infestation == 0 | -0.06 | 0.02 | -3.21 | < 0.01* |

Table A3. Means comparisons of golden eagle nestling corticosterone at each level of *H. inodorus* infestation in nests in southwestern Idaho, USA in 2015. An asterisk (*) indicates a statistically significant difference.

| Comparison | Estimate | Std. Error | z value | Pr(>\|z\|) |
| --- | --- | --- | --- | --- |
| Low infestation - No infestation == 0 | 5.78 | 3.45 | 1.67 | 0.09 |
| High infestation - No infestation == 0 | 25.21 | 5.21 | 4.83 | < 0.01* |
| High infestation - Low infestation == 0 | 19.43 | 6.35 | 3.06 | < 0.01* |

Table A4. Confidence intervals of golden eagle nestling telomere length for each sex at each level of *H. inodorus* infestation in nests in southwestern Idaho, USA in 2016. Telomeres of female nestlings significantly shortened with age in highly infested nests, as shown by confidence intervals that do not overlap zero. The telomeres of female nestlings did not change in nests with either no infestation or low infestation nests. Likewise, male nestling telomere length did not change with infestation level.

| Nestling Sex | Infestation Category | Age Estimate | Standard Error | Degrees of Freedom | Lower Confidence Interval | Upper Confidence Interval |
| --- | --- | --- | --- | --- | --- | --- |
| Male | No infestation | -0.008 | 0.007 | 22.3 | -0.021 | 0.006 |
| Female | No infestation | -0.002 | 0.011 | 15.2 | -0.026 | 0.022 |
| Male | Low infestation | -0.005 | 0.007 | 33.4 | -0.020 | 0.010 |
| Female | Low infestation | -0.009 | 0.006 | 27.3 | -0.021 | 0.004 |
| Male | High infestation | -0.002 | 0.007 | 29.6 | -0.012 | 0.017 |
| Female | High infestation | -0.035 | 0.008 | 28.2 | -0.051 | -0.018 |
